# *In vitro* α–Glucosidase Inhibition, Cytotoxicity, SAR, Swiss ADME Prediction and Molecular Docking Study of New *N*–Substituted Hydantoin Derivatives

**DOI:** 10.1002/open.202400119

**Published:** 2025-02-03

**Authors:** Ndivhuwo R. Tshiluka, Dakalo T. Mbedzi, Mpelegeng V. Bvumbi, Simon S. Mnyakeni‐Moleele

**Affiliations:** ^1^ Research Centre for Synthesis and Catalysis Department of Chemical Sciences University of Johannesburg Kingsway Campus, P.O. Auckland Park 2006 South Africa; ^2^ Department of Biochemistry and Microbiology University of Venda Private Bag X5050 Thohoyandou South Africa; ^3^ Department of Chemistry University of Venda Private Bag X5050 Thohoyandou South Africa

**Keywords:** Hydantoin, α-Glucosidase, Cytotoxicity, Swiss ADME and Molecular Docking

## Abstract

Diabetes is a chronic metabolic disorder affecting about 463 million people globally. α‐Glucosidase (EC.3.2.1.20) inhibitors are among the FDA‐approved oral anti‐diabetic medications used to treat type 2 diabetes. In search of new potential α‐glucosidase inhibitors, fifteen of our previously synthesized hydantoin derivatives **8 a**–**o** were evaluated for their antidiabetic activity. All compounds **8 a**–**o** showed weak α‐glucosidase inhibition at 10, 50 and 100 μM. However, at 200 μM, compound **8 o** was the most potent among the series followed by compounds **8 a, 8 d, 8 l** and **8 n** exhibiting moderate inhibition. The established SAR depended upon the exchange of methyl with methoxy and dioxole derivatives at positions 3 and 4 of the phenyl ring. Cytotoxicity studies revealed that most of the compounds have no cytotoxic effect. Furthermore, Swiss ADME predictions of compounds **8 a**, **8 d**, **8 i**, **8 l** and **8 o** showed high gastrointestinal intestinal absorption required for intestinal absorption of any drug. Most compounds did not obey drug‐likeness character since they violated Ghose and Veber rules with MW>350 and rotors>11. Molecular docking was carried out to investigate the binding interaction of compounds with the active site of α‐glucosidase. The results correlated well with those of the experimental, thereby contributing towards the development of new α‐glucosidase inhibitors.

## Graphical abstract

This study presents the antidiabetic activity of new hydantoin esters **8 a**–**o**. Results revealed that compounds **8 a**–**o** were active against α‐glucosidase at 200 μM. Toxicity study showed that all compounds were nontoxic. ADMET predictions showed that all compounds have a drug‐likeness character. Molecular docking simulations were similar to the experimental results.

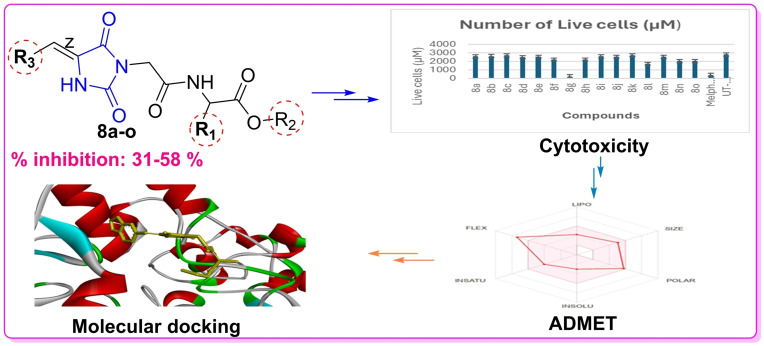

## Introduction

Diabetes is a chronic high blood glucose metabolic disorder that occurs when the pancreas produces insufficient insulin or the body does not recognize the insulin produced.^[1]^ To date, only three types of diabetes are known: type 1 (T1DM), type 2 (T2DM) and gestational diabetes mellitus.^[2]^ In 2019, the prevalence of diabetes worldwide was estimated to be 9.3 % (463 million people) and this figure is expected to increase to 10.2 % (578 million) by 2030 and 10.9 % (700 million) by 2045.^[3]^ The current treatment of T1DM involves the daily injection of insulin whereas T2DM involves using anti‐diabetic medications such as α‐glucosidase inhibitors amongst others.^[4]^

**Figure 1:**
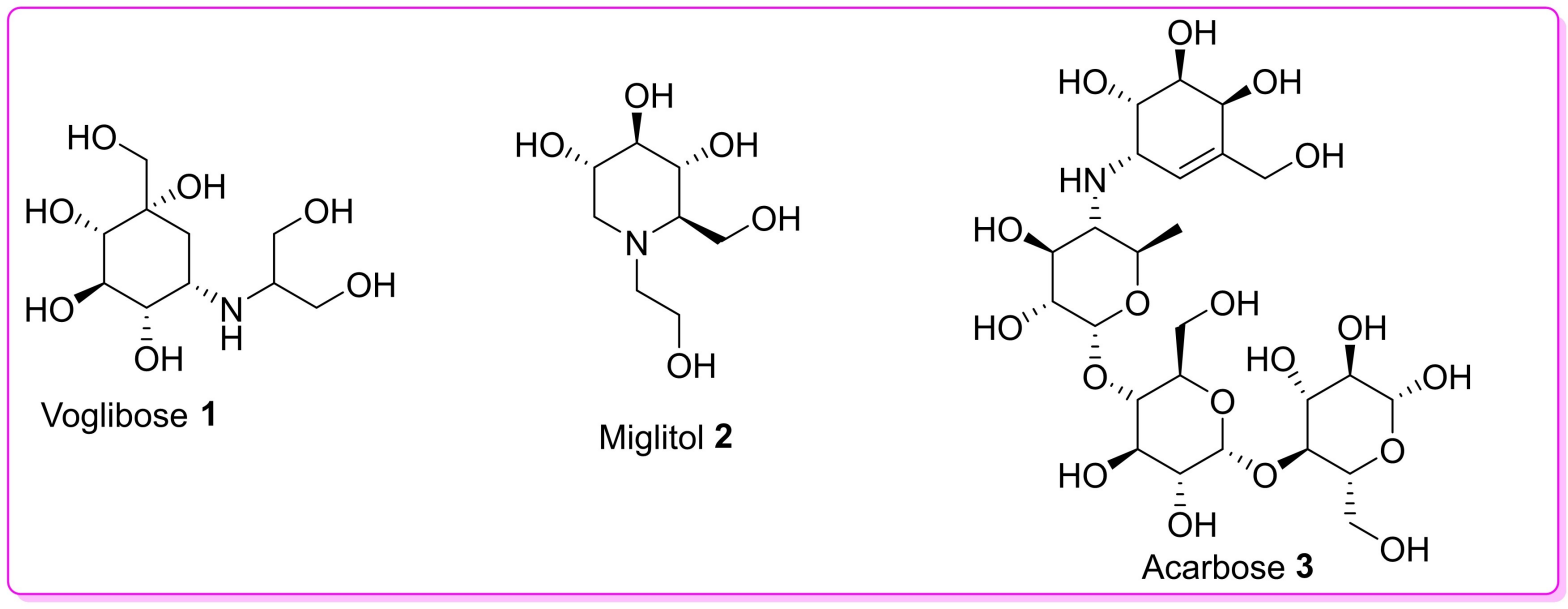
Known α‐glucosidase inhibitors.

α‐Glucosidase (EC.3.2.1.20) inhibitors are a class of oral FDA‐approved anti‐diabetic drugs that are currently used as a treatment for the management of T2DM.^[5]^ Furthermore, if necessary these drugs may be prescribed to patients with T2DM alone or in combination with other anti‐diabetic drugs such as sulfonylureas or biguanides.^[6]^ The primary function of α glucosidase inhibitors is to delay the absorption of glucose brush borders of epithelial cells of small intestines.^[7]^ Mechanistically α glucosidase works by inhibiting the glucose enzymes that convert complex non‐absorbable carbohydrates into simple absorbable carbohydrates.^[8]^ Intestinal absorption can be precisely and accurately predicted using the Swiss ADME web focusing on the compound′s pharmacokinetics, permeability and absorption.^[9]^ Despite considerable efforts made towards the development of new potential α glucosidase inhibitors to treat type 2 diabetes, only three α glucosidase inhibitors are known to date namely: Voglibose **1**, miglitol **2** and acarbose **3**.^[10]^ (Figure 1 **)**.

**Figure 2:**
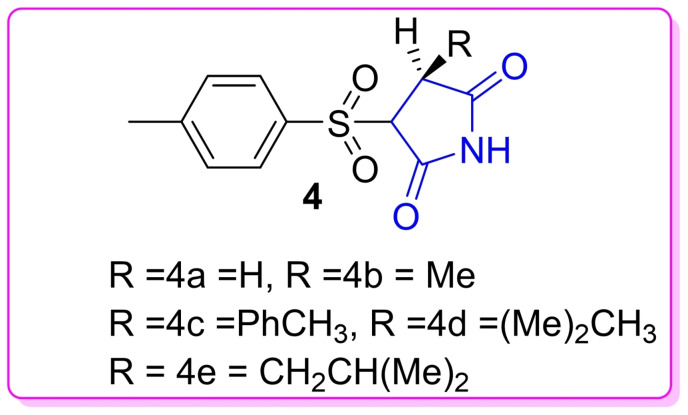
Compounds containing hydantoin moiety with anti‐diabetic properties.

Recently compounds containing hydantoin moiety have been reported to show promising antidiabetic properties.^[11]^ For this reason, more research on hydantoin‐containing compounds is urgently needed in search of new potential α‐glucosidase inhibitors. Hydantoin is a 5‐membered heterocyclic amide with two carbonyls at positions 2 and 4. Furthermore, Hydantoin‐containing compounds have been reported to possess a variety of pharmacological properties such as anti‐microbial^[12]^ anti‐inflammatory^[13]^ anti‐tuberculosis^[14]^ anti‐HIV^[15]^ anti‐malarial^[16]^ as well as anti‐arrhythmic.^[17]^ Ahmad and co‐workers have reported hydantoin‐containing compounds **4** (Figure 2 **)** with anti‐diabetic activity.^[18]^ Previously our research group reported interesting results on the design and synthesis of compounds containing glitazone, rhodanine and hydantoin moieties.[^19^, ^20^, ^21^] We also reported the anti‐diabetic activity of glitazone and rhodanine‐containing compounds.^[20]^ Thus our present paper reports the *in vitro* α‐glucosidase inhibition, cytotoxicity, structure‐activity relationship (SAR) analysis, *in silico* Swiss ADME predictions and molecular docking studies of new hydantoin‐containing compounds **8 a**–**o**.

## Results and Discussion

### Chemistry

**Figure 3:**
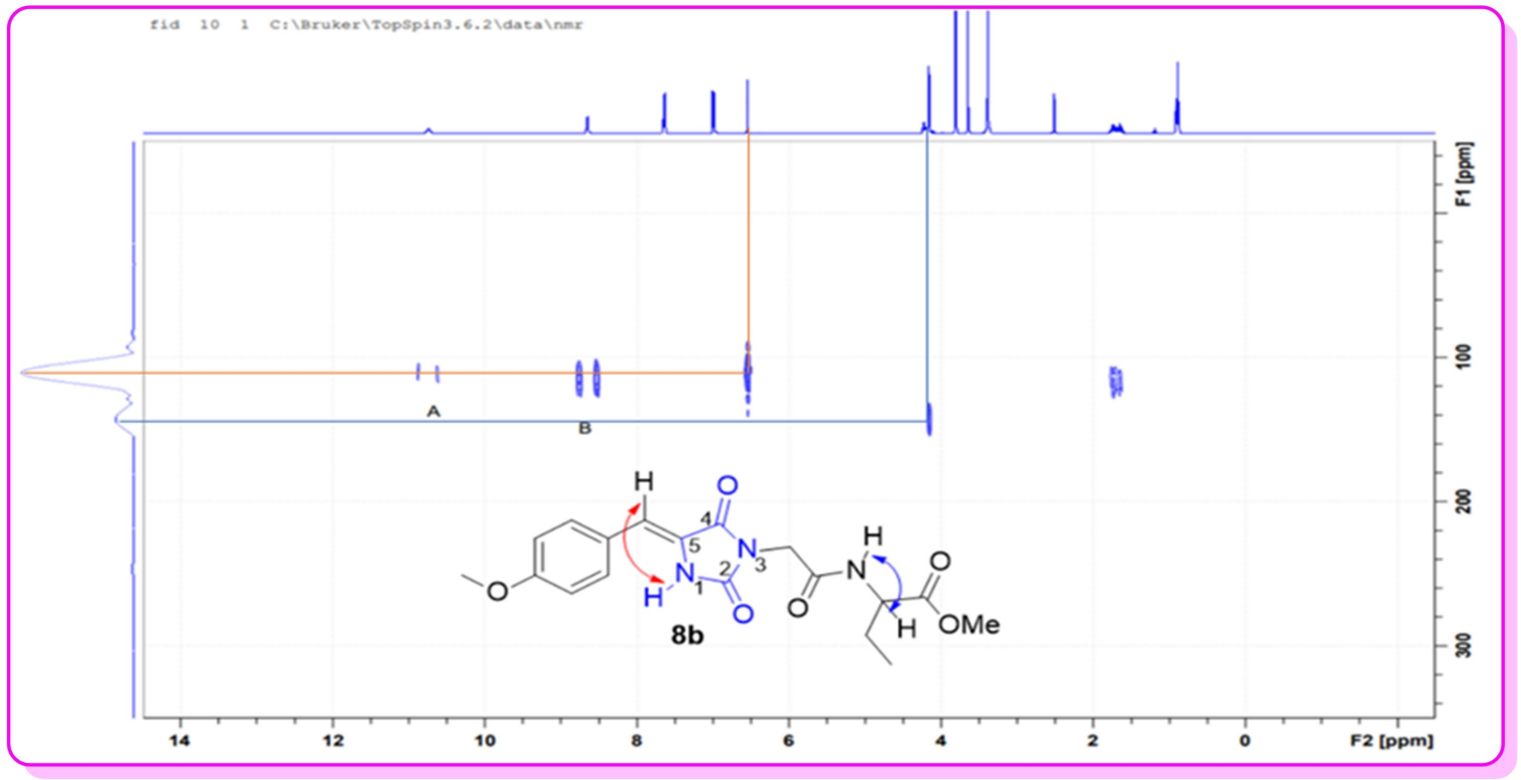
^1^H‐^15^N HMBC of compound **8 b**.

**Scheme 1:**
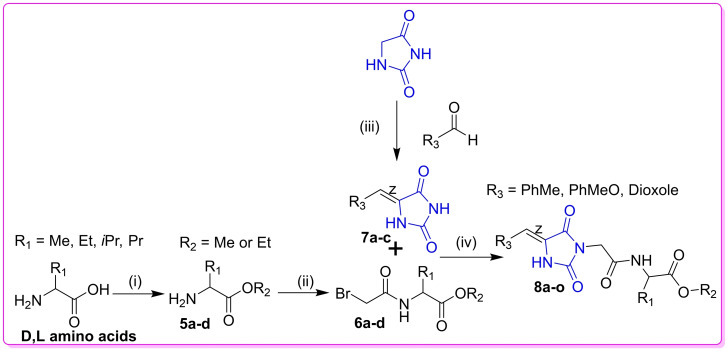
Synthesis of target ethyl or methyl [2‐(5‐benzylidene)‐2,4‐dioxoimidazolidin‐3‐yl] acetyl esters **8 a**–**o: Reagents and Condition**s; (i) MeOH or EtOH, SOCl_2_, reflux, 6 h (ii) Bromo acetyl chloride, DCM/H_2_O, −10 °C, 16 h (iii) Piperidine, reflux, 7 h (iv) MeOH, KOH, reflux, 12 h.

**Figure 4:**
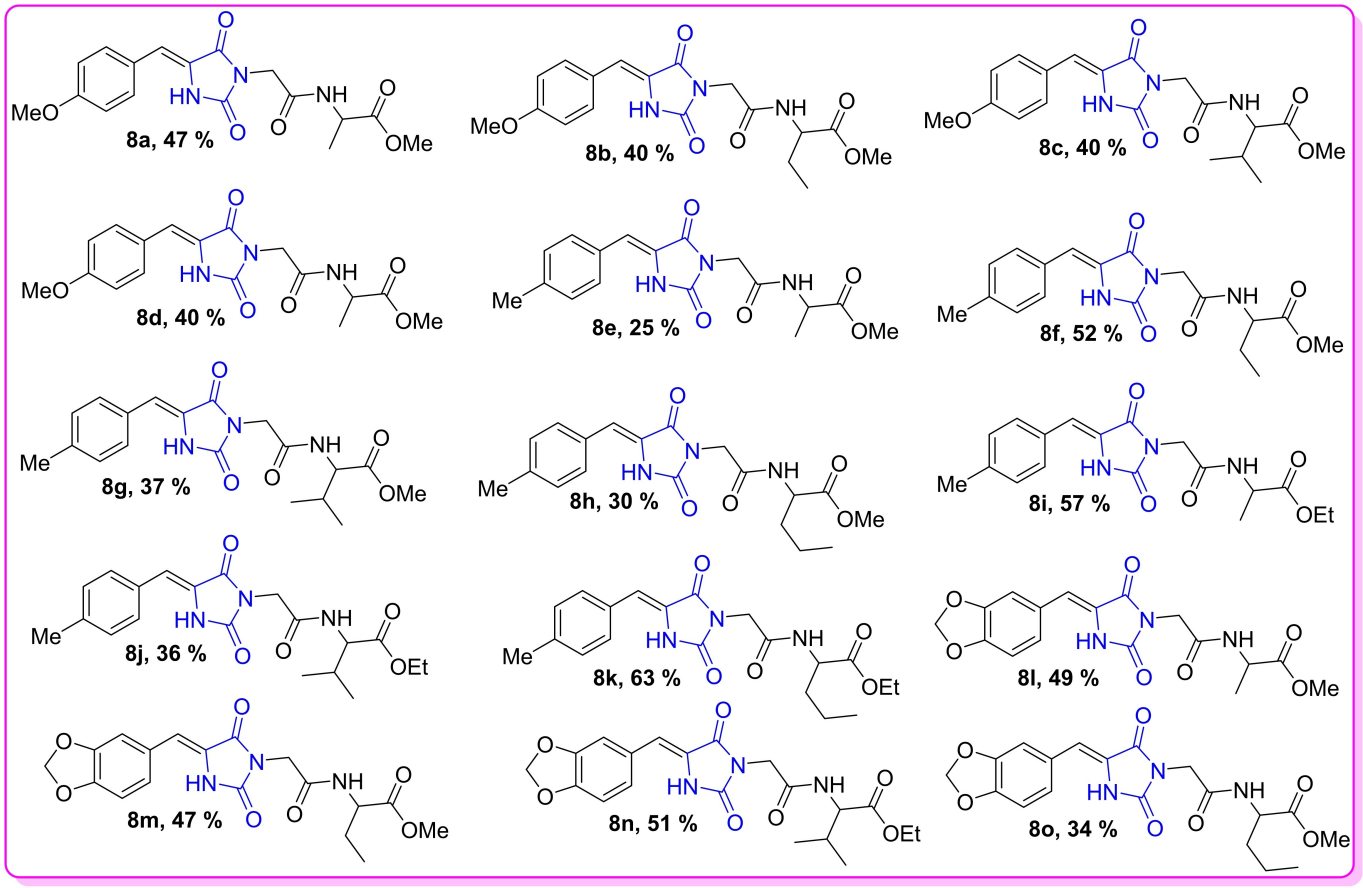
Synthesized hydantoin target compounds **8 a**–**o** and their final respective yields (50 %).

All ethyl or methyl [2‐(5‐benzylidene)‐2,4‐dioxoimidazolidin‐3‐yl] acetylamino esters **8 a**–**o** were obtained as explained from our previous work^[22]^ and their characterization data can be accessed on https://doi.org/10.1177/17475198221104 and in Figure 3. Furthermore, their chemical synthetic route and target structures are shown in Scheme 1 and Figure 4 respectively.

#### ^1^H‐^15^N HMBC Structure Elucidation

Although the ^1^H NMR spectra of the products **8 a**–**o** showed the presence of one N‐H signal at ∼ 10.7 ppm consistent with mono‐N‐alkylation, the long‐range ^1^H−^15^N HMBC experiment was acquired to confirm whether the monoalkylation took place at the N‐1 position or N‐3 position of the hydantoin moiety. Lending credence to the successful mono‐alkylation of these compounds was that ^1^H NMR spectra of compounds **8 a**–**o** showed only one N−H signal appearing at ∼ 10.7 ppm as compared to starting material **7 a**–**c** where the N‐1 signal was observed at ∼ 10.2 ppm and N‐3 was observed at ∼ 11.2 ppm. Furthermore, ^1^H−^15^N HMBC **(**Figure 3 **)** was used to further confirm the regioselectivity of N‐alkylation of hydantoin between the N‐1 and N‐3 positions.

The spectrum in Figure 3 shows that the only possible correlation is between N‐1 and arylidene proton which are four bonds away from one another. This confirmed that nucleophilic acyl substitution of compounds **8a‐o** took place at the more acidic N‐3 position of the hydantoin moiety as there cannot be a correlation of N−H at the 3‐position with arylidene proton since they are more than four bonds away from each other. (Figure 4).

### Biology

#### Cytotoxicity

Cytotoxicity screening is one of the important tools used to determine whether a compound is toxic or not. Furthermore, these assays are performed by testing the ability of cells to continue proliferating in the presence of a test compound or substance over a specific period.^[23]^ All hydantoin title compounds **8 a**–**o** were screened for their *in vitro* cytotoxicity activity using the Caco_2_ cell line against the untreated (UT) and melphalan as a control. Table 1 shows live‐cell data after 48 hours of treatment with 100 μM. Any compound with living cells below 2000 μM was considered to be toxic.

**Table 1:** *In vitro* cytotoxicity and α‐glucosidase evaluation of hydantoin derivatives **8 a**–**o**.

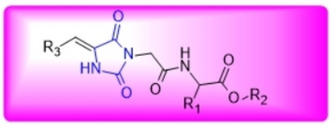								
Compound	R_1_	R_2_	R_3_	Cytotoxicity (μM)	α‐glucosidase Inhibition (50 %)			
				**Live cells**	**10 μM**	**50 μM**	**100 μM**	**200 μM**
**8a**	Me	OMe	OMePh	2605± 293	−	−	−	51.65± 2.92
**8 b**	Et			2643± 311	−	0.43± 16.80	−	39.32± 1.01
**8 c**	*i*Pr			2703± 270	−	3.37± 11.50	9.51± 19.70	41.04± 2.62
**8 d**	Pr			2503± 276	‐	13.10± 16.00	24.30± 29.20	54.21± 1.55
**8 e**	Me	OMe	MePh	2567± 299	‐	9.01± 16.40	9.75± 6.40	31.35± 1.95
**8 f**	Et			2230± 570	4.92± 18.00	8.87± 21.30	12.20± 10.90	‐
**8 g**	*i*Pr			234± 179	1.80± 13.80	4.13± 4.26	18.00± 3.95	45.23± 3.60
**8 h**	Pr			2234± 720	3.45± 13.60	16.30± 15.20	25.40± 7.88	42.76± 4.30
**8 i**	Me	OEt	MePh	2595± 289	‐	‐	‐	44.58± 0.99
**8 j**	*i*Pr			2565± 198	6.49± 17.80	13.80± 9.60	19.20± 10.20	48.47± 2.33
**8 k**	Pr			2730± 213	−	14.50± 5.42	17.00± 8.54	41.94± 3.31
**8 l**	Me	OMe	Piperonyl	1732± 179	‐	7.70± 17.80	9.60± 23.00	57.37± 4.05
**8 m**	Et			2534± 176	‐	6.94± 10.90	11.20± 11.00	37.65± 10.04
**8 n**	i Pr	O Et		2039± 740	0.73± 11.40	14.00± 16.40	28.30± 17.30	57.41 ± 2.79
**8 o**	Pr			2033± 467	11.30± 15.20	16.00± 17.90	37.40± 24.90	57.77 ± 6.38
**Melphalan**	–	–	–	397± 730	‐	‐	‐	‐
**UT**–control	–	–	–	2812± 273	‐	‐	‐	‐
**EGCG**	–	–	–	‐	97.03± 0.41	97.03± 0.41	97.03± 0.41	97.03± 0.41

**Figure 5:**
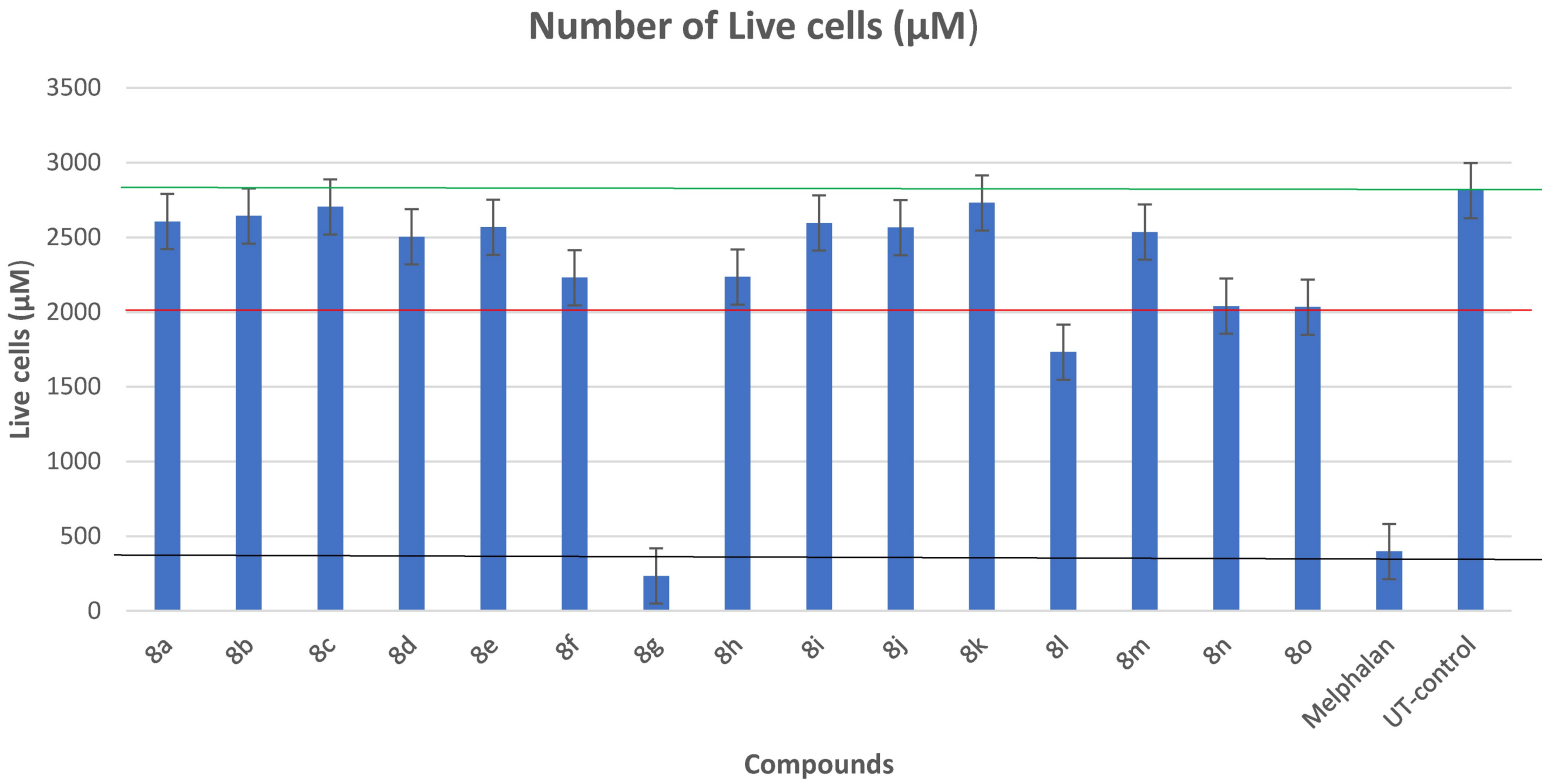
**Cytotoxicity activities of compounds 8 a**–**o tested on Caco2 cells**. Green line: Average live cell number of untreated (UT) control population; Red line: 70 % of average live cell number of untreated (UT) control population; Black line: Average number of live cells treated with 30 μM melphalan (positive control). Error bars indicate the standard deviation of the mean of 4 replicate wells from a single experiment.

Generally, the results in Figure 5 revealed all derivatives **8 a**–**o** did not show any toxicity effect except compounds **8 g** and **8 l** exhibiting live cells of 234 μM and 1732 μM respectively.

The results for 4‐methoxyphenyl hydantoin amino esters **8 a**–**d** showed that all these compounds were not toxic, with activities ranging from 2503 μM to 2703 μM. As can be seen from the graph in Figure 5, the extension of amino esters by exchanging the methyl alanine **8 a** with methyl butanoate **8 b** and methyl vallinate **8 c** has improved the cytotoxicity activity with live cells from 2605, 2643, and 2703 μM, respectively.

With 4‐methylphenyl hydantoin amino esters **8 e**–**k**, only methyl valinate derivate **8 g** was found to be toxic with the activity of 234 μM. The rest of the compounds exhibited activities ranging from 2230 to 2730 μM. The results revealed that increasing the amino ester chain from methyl alaninate **8 h** to methyl butanoate **8 k** has significantly improved toxicity activity with live cells from 2234 μM to 2730 μM. The methyl butanoate **8 l** among the piperonyl hydantoin amino esters **8 l**–**o** was found to be toxic with live cells of 1732 μM.

With these compounds, the results showed that the extension of amino acids from methyl alaninate **8 m** to ethyl butanoate **8 o** has significantly decreased the cytotoxicity activities with live cells decreasing from 2534 to 2033 μM as represented in Figure 5.

#### α–Glucosidase Inhibition

Using Saccharomyces cerevisiae α‐glucosidase enzymes, hydantoin derivatives **8 a**–**o** were evaluated for them for their *in vitro* α‐glucosidase activity. The results are summarized in Table 1. Generally, when compared to the standard reference drug Epigallocatechin gallate (EGCG) which exhibited inhibition of 97.03 %, the majority of compounds had different degrees of α‐glucosidase inhibitory activity, with weak α‐glucosidase inhibitory activity observed from 10, 50 and 100 μM concentration. However, there was a great improvement of α‐glucosidase inhibition at 200 μM where all compounds showed inhibition ranging from 37.40 to 57.77 % as depicted in Table 1. Compounds **8 e**, **8 a**, **8 l**, **8 n** and **8 o** exhibited moderate inhibitory activity with inhibition values of 51.35 %, 51.65 %, 57.37 %, 57.41 % and 57.77 % respectively.

Among the 4‐methoxyphenyl hydantoin amino esters **8 a**–**d,** the results from Table 1 clearly show there **is** no α‐glucosidase inhibition observed at 10 μM. As the concentration was increased to 50 and 100 μM respectively, the extension of amino esters from methyl to butyl affected the α‐glucosidase inhibition. This was evident with methyl valinate **8 c** and methyl butanoate **8 d** which showed some α‐glucosidase inhibition of 3.37–24.30 % as compared to methyl alaninate **8 a** with no activity. At 200 μM, α‐glucosidase inhibition of methyl valinate **8 c** improved to 54.21 % which is the highest in the series followed by methyl alaninate **8 d** exhibiting 51.65 % inhibition.

Within the 4‐methyl phenyl hydantoin amino series **8 e**–**k**, All the derivatives showed no inhibition at 10 μM as shown in Table 1. At 50 and 100 μM, almost all the compounds picked some α‐glucosidase activities ranging from 4.13 % to 25 % except for ethyl alaninate **8 i** which showed no activity. All the derivatives **8 e**–**k** showed significant improvement of α‐glucosidase inhibition at 200 μM ranging from 31.35–44.58 %. The results further revealed that the extension of the amino esters chain had also improved the α‐glucosidase activities at all concentrations.

Concerning piperonyl derivatives hydantoin amino esters **8 l**–**o**, only derivative **8 o** showed minor α‐glucosidase inhibition of 11.30 % at 10 μM and the rest of the derivatives were not active. At 50 μM and 100 μM, all the derivatives **8 l**, **8 m**, **8 n,** and **8 o** displayed some α‐glucosidase inhibition of 9.60 %, 6.94 %, 14.00 % and 37.40 % respectively as shown in Table 1. At the highest concentration of 200 μM, Ethyl butanoate **8 o** exhibited the highest α‐glucosidase inhibition of 57.77 % among all the compounds in all series followed by methyl valinate **8 n** with 57.41 % inhibition. From these results, it was evident that although the majority of compounds did not show excellent α‐glucosidase activities the piperonyl derivatives **8 l**–**o** emerged as a new class of promising α‐glucosidase inhibitors.

#### Structure–Activity Relationship (SAR)

**Figure 6:**
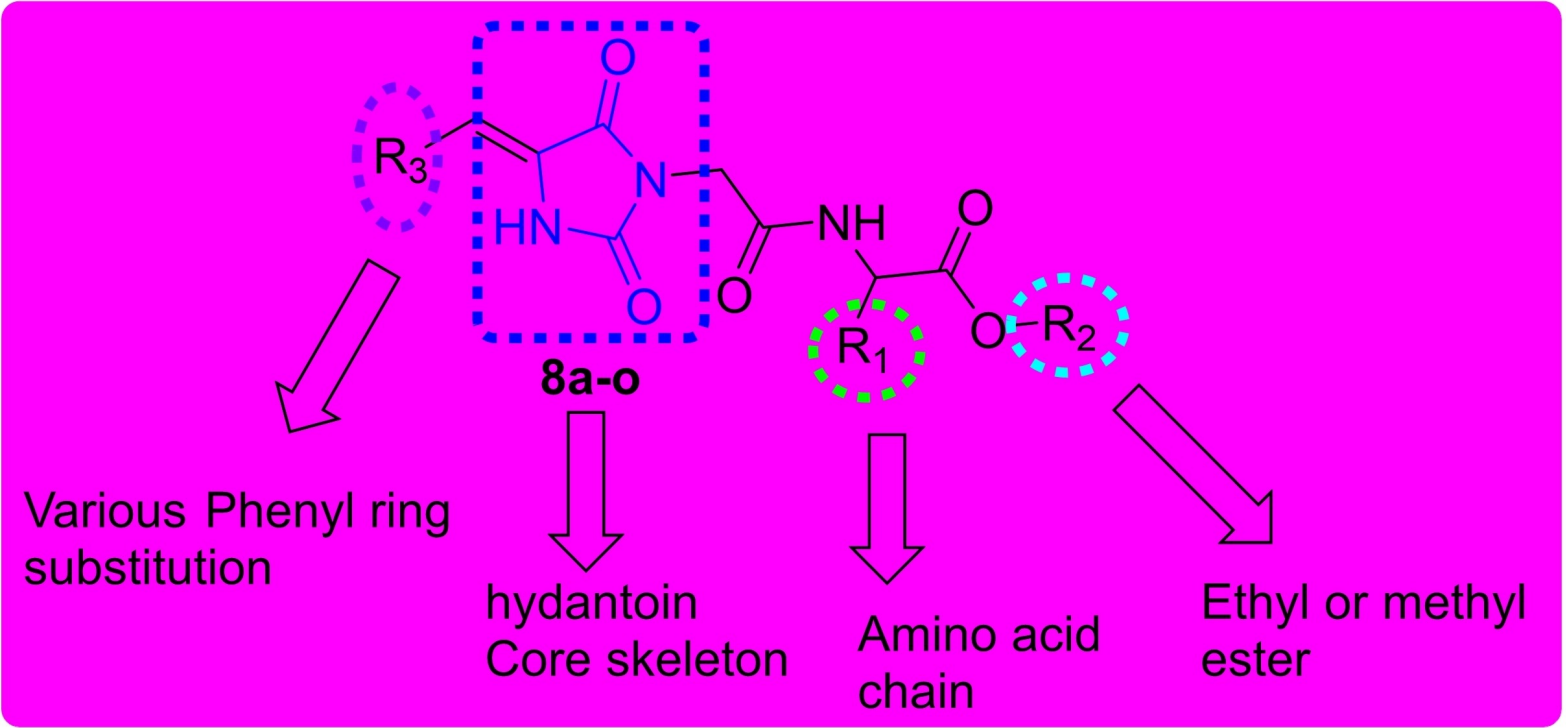
The basic skeleton of hydantoin derivatives **8 a**–**o**

The structure‐activity relationship of all the synthesized hydantoin compounds **8 a**–**o** was established. All the derivatives have the same main skeleton, but they differ from each other via the substitution of the phenyl ring and ethyl or methyl amino esters chain. (Figure 6)

**Figure 7:**
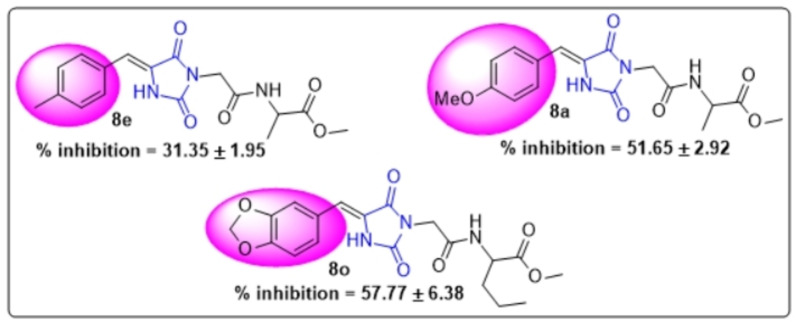
α‐glucosidase inhibition of compounds **8 e**, **8 a** and **8 o** at 200 μM.

The results suggest that the exchange or substitution of either methyl or methoxy phenyl with electron‐withdrawing dioxole derivatives has played a significant role in the α‐glucosidase inhibitory activity. This was evident at 200 μM where dioxole derivative **8 l**–**o** showed an improved inhibition of 37.65 %–57.77 % as compared to methyl or methoxy phenyl derivatives **8 a**–**k** which exhibited low inhibition ranging from 39.32 % to 51.65 % as depicted in Figure 7. These results suggested that the introduction of oxygen deactivates the phenyl ring due to its electron‐withdrawing effects which play a critical role in the α‐glucosidase activity.

**Figure 8:**
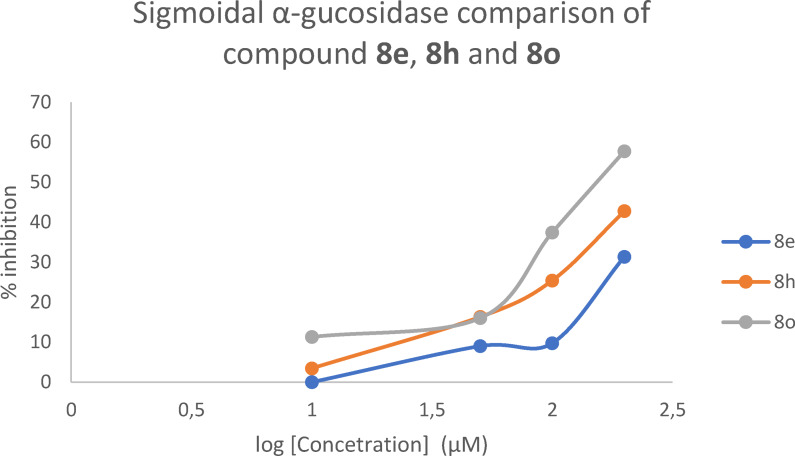
Sigmoidal α‐glucosidase comparison of compounds **8 e**, **8 h** and **8 o**.

To our disappointment, we have noticed the increasing sigmoidal curves for all norvalinate‐containing compounds **8 e**, **8 h** and **8 o** which strongly revealed that the α‐glucosidase inhibition increases as the concentration was increased from 10 μM to 200 μM. (Figure 8) As expected the curves started with a low slope as it approaches towards maximum inhibition of 57.77 %. These results suggested that the extension of the amino acid chain from methyl to butyl has also played a role in the α‐glucosidase inhibitory activity.

#### General Rules for Human Gastrointestinal Intestinal Absorption Classification

Lipinski, Ghose, Veber, and Elgan, Muegge rules are the most widely used classical rules to predict intestinal absorption.^[24]^ According to Lipinski rule,^[25]^ poor absorption and permeation are more likely when the molecular weight (MW) is over 500 Da, lipophilicity (LogP) and hydrogen‐bond donors (HBD) are more than five, and there are more than ten hydrogen‐bond acceptors (HBA). Ghose rule^[26]^ modified to MW of 160 to 480 Da, a LogP of −0.4 to 5.6, a molar refractivity (MR) of 40 to 130, and a total number of atoms (TNA) of 20 to 70. Veber's rule^[27]^ further increases the criteria for bioavailability with less than ten rotatable bonds (RB) and a polar surface area (PSA, Å 2) no greater than 140. Muegge's rule^[28]^ included other parameters to differentiate between drug‐like and nondrug‐like compounds by stricter rules which MW 200–600, LogP from ≤2 to 5, PSA ≤ 150, number of rings (NR) ≤7, number of carbons (NC) > 4, number of heteroatoms (NH) > 1, RB ≤ 15, HBD ≤5, HBA ≤ 10.

#### Physicochemical Property Evaluation

All selected compounds **8 a**, **8 d**, **8 i**, **8 l** and **8 o** were found to comply with Lipinski′s rule when compared to the standard drug ECGC. This proposed that the molecular structure of these compounds is almost the same as that of the oral drugs. The physicochemical properties of all derivatives are within the established values in the ranges of ≤500, ≤5, ≤140 A^2^, and ≤10, respectively corresponding to molecular weight (MW), the number of hydrogen bond donors (nHBD), topological polar surface area (TPSA), logP, and the number of hydrogen bond acceptors (nHBAs) as depicted in Table 2. All compounds presented either 9 or 11 rotatable bonds. The MR was found to be 97.65, 107.27, 100.93 and 102.03 for compounds **8 a**, **8 d**, **8 i**, **8 l** and **8 o** respectively.

**Table 2:** Physiochemical properties of compounds **8 a**–**o** in comparison with the control drug.

Compound	Physiochem Properties	Lipophilicity	Water solubility	Pharmacokinetics	Drug Likeness and Bioavailability	Medicinal Chemistry
**8 a**	Mw=363.37 g/mol; nRT=9 nHBA=6; nHBD=2 MR=97.65 TPSA=114.04 A^2^	Consensus Log (Po/w)=0.60 Log (Po/w) (SILICOS IT)=0.78	Log S (ESOL)= −2.15, Soluble Log S (Ali)=−2.75 Soluble	GI abs=High BBB per=No P‐gb sub=Yes CYP1A2=No CYP2C19=No CYP2C9=No CYP2D6=No CYP3A4=No Bioavail score=0.55	Lipinski=Yes, 0 violation Ghose=N0, WLOGP<0.4 Veber=Yes Egan=Yes Muege=Yes	Pains=0 alert Brenk=1, hydantoin Leadlikeness=No, MW>350, Rotors>7 Synth access=3.24
**8 d**	Mw=391.42 g/mol; nRT=11 nHBA=6; nHBD=2 MR=107.27 TPSA=114.04 A^2^	Consensus Log (Po/w)=1.26 Log (Po/w) (SILICOS IT)=1.58	Log S (ESOL)= −2.74, Soluble Log S (Ali)=−3.68 Soluble	GI abs=High BBB per=No P‐gb sub=Yes CYP1A2=Yes CYP2C19=No CYP2C9=No CYP2D6=No CYP3A4=No Bioavail score=0.55	Lipinski=Yes, violation Ghose=Yes Veber=No, Rotors>10 Egan=Yes Muege=Yes	Pains=0 alert Brenk=1, hydantoin Leadlikeness=No, MW>350, Rotors>7 Synth access=3.50
**8 i**	M w=361.39 g/mol; n R T=9 n H B A=5; n H B D=2 M R=100.93; T P S A=104.81 A^2^	Consensus Log (P o/w)=1.24 Log (P o/w) (S I L I C O S I T)=1.62	Log S (E S O L)= −2.62, Soluble Log S (A l i)=−3.34 Soluble	G I abs=High B B B per=No P‐g b sub=Yes C Y P1 A2=Yes C Y P2 C19=Yes C Y P2 C9=No C Y P2 D6=No C Y P3 A4=No B i o a v a i l=0.55	L i p i n s k i=Yes, 0 violation G h o s e=Yes V e b e r=Yes E g a n=Yes M u e g e=Yes	P a i n s=0 altert B r e n k=1, hydantoin L e a d l i k e n e s s=2, M w>350, R o t o r s>7 S y n t h a c c e s s=3.50
**8 l**	M w=391.38 g/mol; n R T=9 n H B A=7; n H B D=2 M R=102.03; T P S A=123.27 A^2^	Consensus Log (P o/w)=0.86 Log (P o/w) (S I L I C O S I T)=1.00	Log S (E S O L)= −2.45, Soluble Log S (A l i)=−3.16 Soluble	G I abs=High B B B per=No P‐g b sub=Yes C Y P1 A2=No C Y P2 C19=No C Y P2 C9=No C Y P2 D6=No C Y P3 A4=No B i o a v a i l score=0.55	L i p i n s k i=Yes, 0 violation G h o s e=No, W L O G<0.4 V e b e r=Yes E g a n=Yes M u e g e=Yes	P a i n s=0 alert B r e n k=1 alert, hydantoin L e a d l i k e n e s s=No, M W>350, R o t o r s>7 S y n t h access=3.70
**8 o**	Mw=419.43 g/mol;nRT=11 nHBA=7;nHBD=2 MR=111.6;TPSA=127.27 A^2^	Consensus Log (Po/w)=1.47 Log (Po/w) (SILICOS IT=1.80	Log S (ESOL)= −3.03,Soluble Log S (Ali)=−4.07 Soluble	GI abs=High BBB per=No P‐gb sub=Yes CYP1A2=No CYP2C19=No CYP2C9=No CYP2D6=No CYP3A4=Yes Bioavail score=0.55	Lipinski=Yes,0 vialotion Ghose=Yes Veber=No,Rotors>10 Egan=Yes Muege=Yes	Pains=0 alert Brenk=1,hydantoin Leadlikeness=No,MW>350,Rotors>7 Synth access=3.96
**EGCG**	Mw=458.37; nRT=4 nHBA=8; nHBD=11 MR=112.06; TPSA=197.37 A^2^	Consensus Log (Po/w)=1.01 Log (Po/w) (SILICOS IT)=0.57	Log S (ESOL)= −3.58, Mod Soluble Log S (Ali)=−4.91 Mod Soluble	GI abs=Low BBB per=No P‐gb sub=No CYP1A2=No CYP2C19=No CYP2C9=No CYP2D6=No CYP3A4=No Bioavail score=0.17	Lipinski=No, NorO>10, NHorOH>5 Ghose=Yes Veber=No, TPSA>140 Egan=NO, TPSA>131.6 Muege=3, TPSA>150, HAcc>10, Hdon>5	Pains=1, catechol A Brenk=1, catechol Leadlikeness=NO, MW>350 Synth access=4.20

#### Lipophilicity and Water Solubility

The lipophilicity partition Po/w values of compounds **8 a**, **8 d**, **8 i**, **8 l** and **8 o** were found to be ranging from 0.60–1.47 when compared with the reference EGCG with Po/w of 1.01. All compounds were predicted to be soluble in water with log S ranging from −2.75 to −4.07 as shown in Table 2 suggesting that they might have the potential ability to cross the blood‐brain barrier.

#### Pharmacokinetics

**Figure 9:**
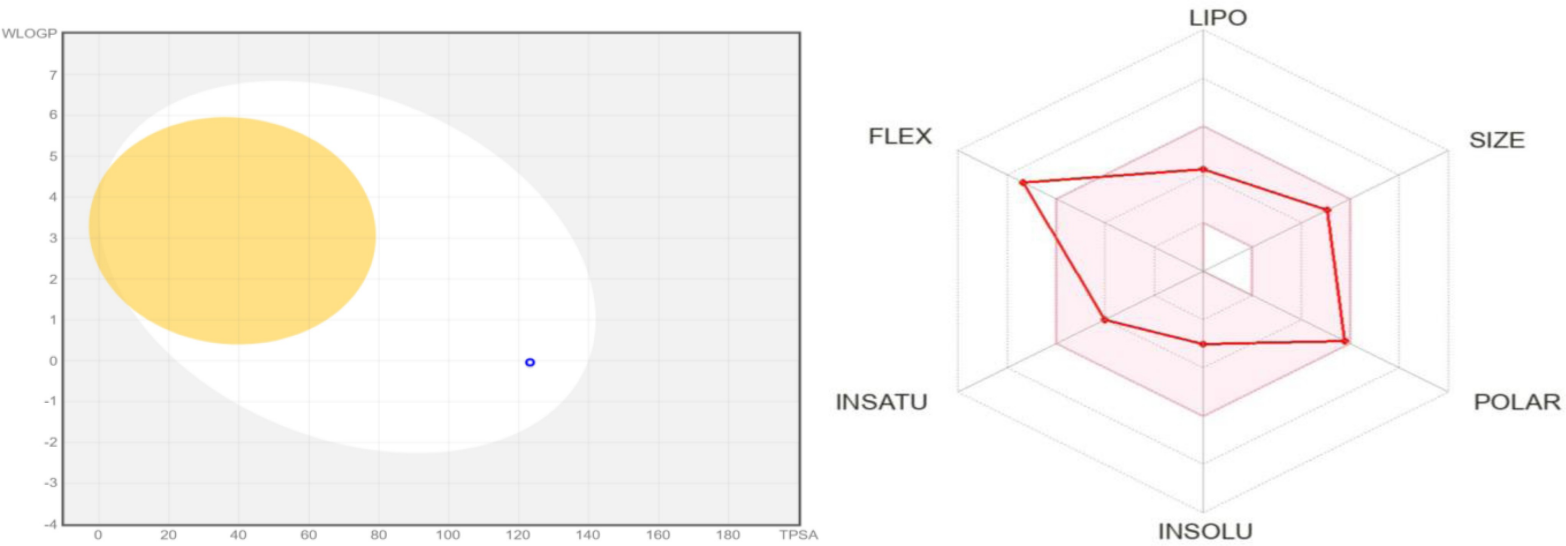
The boiled egg and physiochemical radar chart of compound **8 o**

Pharmacokinetics is one of the most important therapeutic strategies that is used to measure the effectiveness of a drug. Through this strategy, researchers can study the pharmacokinetic characteristics of any compound that has the potential to impact a drug's pharmacological profile.^[29]^ The study uses predictions for gastrointestinal absorption (GI) and blood‐brain barrier (BBB) permeation which can also be represented by the BOILED‐Egg model. For this study, the predictions showed all the selected compounds displayed high GI absorption as can be seen from Table 2 and this is also represented BOILED‐Egg model in Figure 9. In summary, this suggests that these compounds are suitable for human intestinal absorption. With regards to drug metabolism, only **8 d** and **8 i** were found to be CYP1A2 inhibitors whereas compound **8 o** only inhibited the CYP3A4 enzyme.

#### Drug Likeness and Medicinal Chemistry

As shown in Table 2, only compound **8 i** did not violate five drug‐likeness approaches (Lipinski, Muegge, Ghose, Veber, and Egan). The rest of the compounds have violated the Ghose rule with the partition Po/wo values for WLOG less than 0.4 and Veber with rotational bonds exceeding 10. All the selected compounds **8 a**, **8 d**, **8 i**, **8 l** and **8 o** had no pain alert whereas hydantoin moiety was alerted by Brenk structural alert. Furthermore, the compounds presented molecular weight greater than 350 with simple synthetic accessibility scores ranging from 3.24–3.96 suggesting that they have a less lead‐like drug capability.

#### Molecular Docking

Molecular docking studies were carried out to elucidate the interactions and the binding modes between the active site of an α‐glucosidase target (PDBQT) and PPAR‐γ. The selected hydantoin derivatives **8 a, 8 b, 8 e, 8 h, 8 l, 8 m** and **8 o** were evaluated for their α‐glucosidase inhibitors and PPAR‐γ binding activities. The structures of these compounds were visualized and calculated by PyRx using the Discovery Studio Visualizer‐2021 version. With regards to α‐glucosidase inhibition, the docking results clearly show that the binding affinities of these ligands correlate with their experimental *in vitro* α‐glucosidase activity values. The binding affinity of compounds **8 a**–**o** ranged from −6.1 to −6.7 kcal/mol as compared to the recommended standard EGCG (−9.4 kcal/mol) as depicted in Table 2. Interestingly, piperonyl derivatives **8 l**, **8 m** and **8 o** showed an increased binding affinity of −6.4, −6.6 and −6.7 kcal/mol respectively.

**Figure 10:**
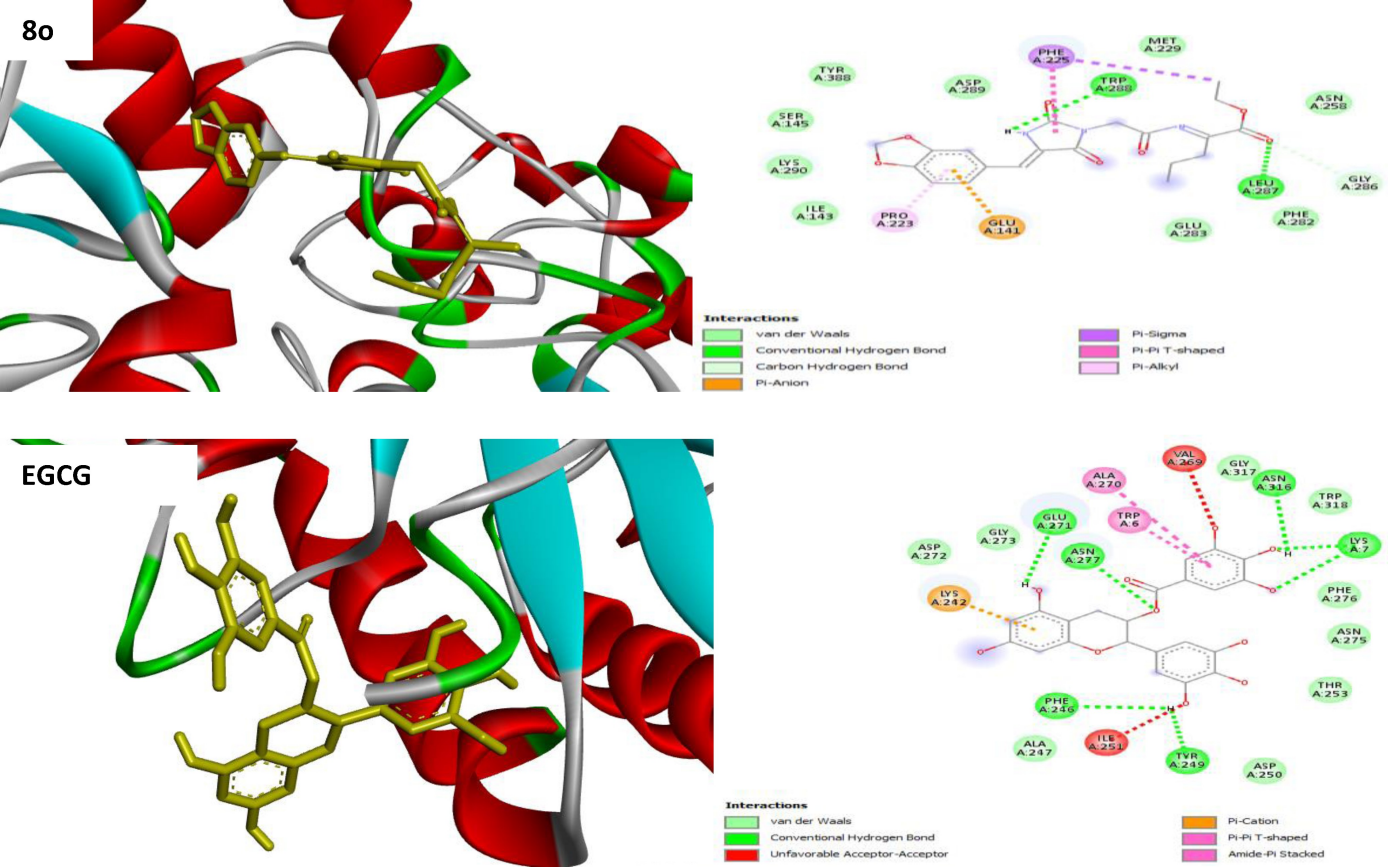
Docking pose of the compound **8 o** and **EGCG** standard in the active site of α‐glucosidase enzyme.

2D interactions of selected compounds well as recommended α‐glucosidase standard (EGCG) with the binding site of ligand‐receptor interaction were also shown in Figure 10. Among the series, piperonyl norvalinate **8 o** was found to the best fit in the binding pocket of the α‐glucosidase enzyme having a docking score binding affinity of −6.6 kcal/mol. Furthermore, compound **8 o** showed good interactions with π‐sigma, π‐π, π‐anion and π‐alkyl with nucleotides PHE 225, GLU 141 and PRO 223 respectively. Moreover, compound **8 o** showed TRP 288 hydrogen bond interaction with the N‐H group of the hydantoin moiety whereas another hydrogen bonding was formed between nucleotide LEU 287 and the carbonyl group of the amino acid moiety.

Methyl‐phenyl alaninate **8 e** was found to have a binding affinity of −6.5 kcal/mol correlating well with its lowest experimental α‐glucosidase inhibition of 31.35 % at 200 μM. In addition, compound **8 e** showed four interactions with nucleotides GLU 143, SER 142, LYS 395 and LYS 290 as shown in Figure 10. GLU 143 formed carbon‐hydrogen bonding with the methylene group of the acetyl moiety and the methoxy group of the amino acid. On the other hand, SER 142 created hydrogen bonding interaction with the carbonyl group of the amino acid. LYS 395 formed hydrogen bonding with the oxygen atom of the amino acid while LYS 290 formed π‐alkyl interaction with hydantoin moiety and carbon‐hydrogen bonding interaction with the carbonyl group of acetyl moiety.

When it comes to PPAR‐γ binding activities, The binding affinity of all selected compounds was higher ranging from −7.0 kcal/mol to −7.8 kcal/mol when compared to the rosiglitazone standard which had a binding affinity score of −6.8 kcal/mol (Table 3). Piperonyl derivatives **8 l**, **8 m** and **8 o** exhibited the highest binding affinity in the range of −7.8, to 7.0 kcal/mol when compared to the *para* methyl and methoxy derivatives **8 a**, **8 b**, **8 e**, **8 h** with binding affinity of −7.2, to 7.5 kcal/mol. Among the piperonyl series **8 l**–**o**, the piperonyl butanoate **8 m** was the highest active derivative with a binding affinity of −7.8 kcal/mol followed by piperonyl alaninate **8 l** and piperonyl norvalinate **8 o** with binding affinity of −7.6 and 7.8 kcal/mol. Surprisingly in this enzyme, the piperonyl novalinate **8 o** was found to be the lowest active derivative with −7.0 kcal/mol binding affinity when compared to all the selected compounds.

**Table 3:** Interaction of selected compounds with α ‐ glucosidase target (PDBQT) inhibitors.

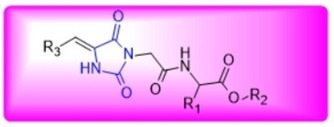					
Compound	_R1_	R_2_	R_3_	Binding affinity (Kcal/mol)	Interactive amino acids or nucleotides
**8 a**	Me	Me	MeOPh	−6.6	MET A:229 van de Waals force
**8 b**	Et			−6.2	LYS A:290 unfavorable positive‐positive; conventional hydrogen bonding, pi alkyl; LYS A:395 pi anion, pi alkyl; LEU A:219, SER A:245 conventional hydrogen bonding; TYR A:388 pi‐pi T‐shaped; GLU A:141 salt bridge
**8 e**	Me	Me	PhMe	−6.5	GLU A:141, SER A:144, LYS A:395 conventional hydrogen bonding; salt bridge; LYS A:290 pi alkyl; conventional hydrogen bonding
**8 h**	Me	Et		−6.7	PHE A:225 pi alkyl; LYS A:290 pi alkyl; PRO A:223 pi alkyl; GLY A:141 salt bridge; ILE A:391 alkyl
**8 l**	Me	Et	piperonyl	−6.7	LYS A:290 pi alkyl; TRP A:288, LEU A:287; conventional hydrogen bonding; PHE A:225 pi sigma; pi‐pi T‐shaped; GLU A:141 pi anion; PRO A:223 pi alkyl; GLU A:141 pi anion; conventional hydrogen bonding
**8 m**	Et			−6.4	ASN A:120 conventional hydrogen bonding; ASP A:124 pi anion, salt bridge, conventional hydrogen bonding; ASN A:171 conventional hydrogen bonding; ARG A:123 pi alkyl
**8 o**	Pr	Me		−6.6	PHE A:225 pi sigma, pi‐pi T‐shaped; TRP A:288 conventional hydrogen bonding; LEU A:287 conventional hydrogen bonding; GLU A:141 pi anion; PRO A:223 pi alkyl
**EGCG**	‐	‐	‐	−9.4	LYS A:242 pi cation; GLU A:272, ASN A:277, ASN A:316, TRP A:6, ALA A:270, Pi‐Pi T shaped; LYS A:7, PHE A:246, TYR A:249 conventional hydrogen bonding; VAL A:269, ILE A:251 unfavorable acceptor

The same compound **8 o** formed an attractive charge on nucleotides TYR 505 on the amide whereas nucleotides LEU 504 formed conventional hydrogen bonding with the carbonyl moiety of the amino acid. Furthermore, nucleotide HIS 482 formed conventional hydrogen bonding with acetyl moiety whereas MET 491 nucleotides formed Pi alkyl bond with Pi bond of benzene moiety. LYS 485 formed pi alkyl bond with Benzene together with both nucleotides LEU 393 and LEU 481 forming pi sigma with the same benzene moiety as shown in Figure 10. With 4‐methyl or methoxy phenyl derivatives **8 a**–**h**, 4‐methoxy butanoate **8 b** was found to be the highest active derivative with a binding affinity of −7.5 kcal/mol followed by 4 methyl alaninate **8 e** with the lowest binding of −7.2 kcal/mol.

## Conclusion

In this study, we have presented α‐glucosidase inhibition, cytotoxicity, SAR, *In silico* Swiss ADME predictions and molecular docking study of our previously synthesized hydantoin derivatives **8 a**–**o** in comparison with EGCG standards. There was a good correlation between experimental α‐glucosidase inhibition and molecular docking. SAR effect depended on the exchange on the extension of amino acids and compounds with electron‐withdrawing oxygen on the phenyl ring were more capable of inhibiting α‐glucosidase activity at 200 μM than those with electron‐donating character. Analysis of the binding mode showed that the compounds were more active on PPA‐γ than on the α‐glucosidase enzyme. Furthermore, it was observed that the exchange of methyl at position 4 with methoxy and dioxole derivatives plays a critical role in the activity profile. *In silico*, Swiss ADME predictions of selected compounds showed poor pharmacokinetic and favorable drug‐likeness character. The GI absorption for all compounds was found to be high suggesting that they are suitable for intestinal absorption. Furthermore, cytotoxicity studies showed that most compounds do not exhibit cytotoxicity activity. Furthermore, these results suggest that this class of compounds has the potential for the development of novel lead therapeutics for treating T2DM.

## Experimental Section

### General

All the required starting materials and reagents were obtained from Sigma‐Aldrich (St. Louis, MO, USA), GE Healthcare Life Sciences (Logan, UT, USA), Lonza (Wakersville, MD, USA) and other commercial suppliers. The characterization techniques of final compounds **8 a**–**o** were described in our previous methodology.^[22]^

### Chemistry

General methods for synthesis of compounds **5 a**–**d**, **6 a**–**d**, **7 a**–**c** and **8 a**–**o**. All the intermediates **5 a**–**d**, **6 a**–**d**, **7 a**–**c** and title compounds **8 a**–**o** were synthesized according to the previously reported method.^[20]^ In addition, the stereochemistry of amino acids **5 a**–**d** used as starting materials was racemic (D, L) whereas compounds **7 a**–**c** were assigned Z‐configuration after comparing them with those previously reported in the literature.[^30^, ^31^]

### Biology

#### Cytotoxicity Activity Assay Protocol

The human colorectal adenocarcinoma cell line (CaCO_2_) used to screen the cytotoxicity of compounds **8 a**–**o** was based on our initially described protocol.^[21]^ According to this method, test compounds **8 a**–**o** were reconstituted in dimethyl sulfoxide (DMSO) to give a final concentration of 100 μM. Samples were sonicated if solubility was a problem and stored at 4 °C until required. Melphalan (100 mM stock) was prepared in DMSO and used as a positive control at a final concentration of 30 μM. The cells (4000 cells/well) were seeded in 96 well plates at 100 μL aliquots and were left for 24 hours to attach. After 24 hours, the 100 μL of the medium was treated with 100 μL aliquots of 200 μM dilution. Cells were maintained in 10 cm culture dishes in 10 mL complete medium (DMEM‐LG+10 % FBS+1× penicillin/streptomycin) and incubated for 48 hours at 37 °C, 5 % CO_2_, and 100 % relative humidity until needed. After incubation 100 μL staining solution Hoechst (5 μg/mL) and 10 μL PI solution were stained on the plates before they were incubated again for 30 min. Quantification of live and dead cells was performed using the ImageXpress Micro XLS Widefield Microscope (Molecular Devices) using a 10× Plan Fluor objective and DAPI and Texas Red filter cubes. Acquired images were analyzed using the MetaXpress software and Multi‐Wavelength Cell Scoring Application Module. Acquired data was transferred to an Excel spreadsheet and data was analyzed and processed.

#### α–Glucosidase Inhibitory Assay Protocol

The α‐glucosidase inhibitions of compounds **8 a**–**o** were screened as described in our previous method.^[21]^ Epigallacatechin gallate (EGCG) was used as positive control. The α‐glucosidase enzyme (Saccharomyces Cerevisiae, 50 μg/mL) and substrate (*p*‐nitrophenyl glucopyranoside) were dissolved in a solution of potassium phosphate buffer (pH 6.3, 3 mM). Final compounds **8 a**–**o** were dissolved in DMSO to give a final concentration of 200 μM. The resultant samples were sonicated to address solubility and stored at 4 °C until required and tested at various concentrations (10, 50, 100 and 200 μM). All samples (5 μL), enzyme (20 μL), and potassium phosphate buffer (60 μL) were added to the 96‐well plate and incubated at 37 °C for 5 min. Thereafter, the substrate (10 μL, 4 mM) was added to the mixture and incubated at 37 °C for 20 min followed by the addition of sodium carbonate (25 μL). Finally, the absorbance readings were measured by BioTek® PowerWave XS spectrophotometer (Winooski, VT, USA). Since the enzyme and substrate controls were not included, the percentage of α‐glucosidase inhibition was calculated using follows:

(1) $$\%\;\;\alpha \text{-}\mathrm{glucosidase}\;\mathrm{inhibition}=\frac{\left(A405nm\;of\;control-\;A405nm\;of\;test\;sample\right)}{\begin{array}{c} A405nm\;of\;control \end{array}}\;\times \;100$$

#### Swiss ADME Method

The Swiss ADME database was used to evaluate the physicochemical properties, pharmacokinetic profile, drug similarity, and medicinal chemistry of all selected compounds, including their lipophilicity and water solubility. The 2D structures from the database were converted into a string‐based search format in order to allow efficient screening and to analyze potential drug candidates.

#### Molecular Docking–Based Virtual Screening Protocol.

Molecular docking virtual screening analysis of the selected hydantoin derivatives **8 a**, **8 b**, **8 e**, **8 h**, **8 l**, **8 m** and **8 o** was performed using PyRx software. *Saccharomyces Cerevisi*a was converted into Autodock structure (PDBQT) file format for analysis using the PyRx software. The grid box dimensions were set to cover the surface area of the enzyme (x, y, and z axes, respectively) and the binding pockets were set to default mode. Before carrying out virtual screening with the selected ligands, all ligands were drawn using ChemSketch and converted to PDBQT using PyMOL software. The best docking poses calculated with PyRx (with the lowest binding energy) of the co‐crystallized ligand complexed with α‐glucosidase were selected to perform the molecular docking analyses. The 2D and 3D analyses of the interaction patterns in protein‐ligand complexes were performed and visualized using the Discovery Studio Visualizer‐2021 version. In all these analyses, the default cut‐off interaction distances were used.

## Conflict of Interests

The authors declare that they have no known competing financial interests or personal relationships that could have appeared to influence the work reported in this paper.

1

## Data Availability

Data will be available on request.
